# Epidemiological analysis of respiratory pathogen detection in 11 regions of Yunnan from 2021 to 2023

**DOI:** 10.3389/fpubh.2026.1771339

**Published:** 2026-02-18

**Authors:** Wenlu Wang, Mei Liu, Xiaolin Zhu, Xianyao Yang, Yang Liu, Yaowu Chen

**Affiliations:** 1 Department of Emergency Medicine, Lijiang People’s Hospital, Lijiang, China; 2 Yunnan Kunming Medical University Precision Medicine Science and Technology Research Institute Co., Ltd./Yunnan Kunming Medical University Precision Medicine Research Institute, Kunming, China

**Keywords:** epidemiological characteristics, infection dynamics, respiratory pathogens, seasonal trends, Yunnan Province

## Abstract

**Objective:**

Acute respiratory infections (ARI), caused by various pathogens, are a significant global public health issue, and their epidemiological characteristics across different populations and regions still require indepth research. Based on laboratory surveillance data, this study analyzed the detection and distribution characteristics of ARI pathogens in 11 areas of Yunnan Province from 2021 to 2023.

**Methods:**

A total of 7,906 ARI patient samples from 11 regions in Yunnan Province were included, and pathogen detection was performed using targeted next-generation sequencing (TNGS) technology covering viral, bacterial, and fungal respiratory pathogens. Stratified analysis was conducted by age group, season, and geographical region, with further evaluation of co-detection patterns.

**Results:**

The results showed that the pathogen detection rate was highest in preschool children (0–5 years group) at 60.59%, with *Streptococcus pneumoniae* (12.56%), Respiratory syncytial virus (6.43%), and *Haemophilus influenzae* (5.87%) being the most common. Seasonal analysis indicates that summer is the peak detection period (31.56%), while the distribution of pathogens varies significantly across different regions, with *Streptococcus pneumoniae* and *Haemophilus influenzae* being the dominant pathogens. The co-detection pattern shows that *Streptococcus pneumoniae* (23.57%) and Human herpesvirus 7 (HHV-7, 10.41%) are the most common co-detected pathogens.

**Conclusion:**

In summary, ARI in Yunnan Province exhibits distinct age specificity, seasonality, and regional differences, with children and summer being the key focuses for prevention and control. These results provide epidemiological evidence for optimizing the prevention and clinical management strategies of ARI in Yunnan Province.

## 1 Introduction

Acute Respiratory Infections (ARI) are one of the most common infectious diseases worldwide, significantly impacting public health, particularly leading to severe illness and complications among children, older adults, and immunocompromised individuals (1–5). Respiratory pathogens are diverse, primarily including viruses, bacteria, and fungi, with influenza virus, respiratory syncytial virus, and *S. pneumoniae* being common pathogens (6). ARI not only poses a threat to individual health but also imposes a heavy burden on the global public health system (7, 8).

In recent years, with the rapid development of molecular biology technologies, particularly the application of highly sensitive and specific detection techniques such as Polymerase Chain Reaction (PCR) and TNGS, the rapid and accurate detection of respiratory pathogens has become possible (9). This has contributed to optimizing diagnosis, treatment, and public health prevention and control measures. However, since the outbreak of the COVID-19 pandemic, the implementation of public health measures (such as mask-wearing and social distancing) may have altered the epidemic patterns of other respiratory pathogens (10). Studies have shown that the occurrence of ARI exhibits distinct age, seasonal, and geographic distribution characteristics (11). However, the Yunnan region has unique geographical characteristics with higher altitude, lower latitude, and stronger ultraviolet radiation. Currently, there are relatively few studies on the epidemiological features of ARI pathogens across Yunnan Province as a whole, and data are lacking. Research has mainly focused on limited areas such as Kunming, Dali, Zhaotong, Qujing, and Yuxi, resulting in relatively limited regional coverage. In the Dali region, loop-mediated isothermal amplification (LAMP) and magnetic particle chemiluminescence methods were employed for pathogen detection, while Zhaotong conducted research based on targeted high-throughput sequencing technology. Other regions predominantly utilized real-time quantitative PCR (RT-qPCR) (12–19). Significant variations exist among studies regarding detection techniques, pathogen coverage, and study populations, resulting in insufficient regional representativeness and comparability of outcomes in current evidence. This makes it challenging to provide an adequate scientific basis for formulating systematic and precise ARI public health prevention and control strategies at the provincial level in Yunnan.

This study is based on the detection data of ARI from 11 regions in Yunnan from 2021 to 2023, analyzing the distribution characteristics of pathogens across different ages, seasons, and regions, exploring the co-detection patterns of pathogens and their epidemic trends, to provide a theoretical basis for the prevention and control measures of ARI in Yunnan Province.

## 2 Methods

### 2.1 Study participants

Data on the detection of respiratory pathogens from outpatients and inpatients in some hospitals in Yunnan Province from January 2021 to October 2023 were collected, encompassing individuals of all age groups. These samples were sent to the laboratory of Yunnan Kunyida Precision Medical Technology Research Institute Co., Ltd. for unified testing. The inclusion criteria for the study subjects were set as follows: presence of fever symptoms accompanied by at least one respiratory symptom, such as cough, nasal congestion, runny nose, sore throat, sputum production, or headache, along with a record of respiratory pathogen testing. The exclusion criteria were as follows: individuals with incomplete clinical records and those who underwent repeated pathogen testing. Additionally, cases with unclear diagnostic results were not included in the data analysis. Since this study is a non-interventional, observational, retrospective investigation that does not involve any interventions in clinical management and only entails the collection of patients’ basic clinical data, it is exempt from the requirement of informed consent. Throughout the research process, the relevant guidelines of the Declaration of Helsinki were strictly adhered to, and stringent confidentiality measures were implemented for patient data.

### 2.2 Sample collection and pathogenic detection

Within 24 h after patient enrollment, respiratory clinical specimens were collected following standardized operating procedures. Sterile swabs were used to sample both tonsils and the posterior oropharyngeal wall separately, with immediate placement into sterile collection tubes for preservation. The included clinical specimen types comprised throat swabs, sputum, and bronchoalveolar lavage fluid (BALF). The total nucleic acids of viruses and bacteria in the samples were extracted using the nucleic acid extraction kit (KS118-BYTQ-24, Guangzhou KingCreate Biotechnology Co., Ltd., China) in conjunction with the automated nucleic acid extraction instrument (KingFisher™ Flex, Thermo Fisher Scientific, United States), strictly following the reagent instructions and instrument operation manual. After extraction, the nucleic acid concentration was quantified using the Qubit dsDNA HS Assay Kit (Invitrogen, United States). After nucleic acid extraction or purification, TNGS was employed for multiplex respiratory pathogen detection. The TNGS assay was performed using the Respiratory Pathogen TNGS Detection Premix (KS608-100HXD96, Guangzhou KingCreate Biotechnology Co., Ltd., China), a method based on the principle combining multiplex PCR amplification with high-throughput sequencing. The brief procedure is as follows: The extracted nucleic acids are first reverse transcribed into cDNA, followed by multiplex PCR amplification targeting highly conserved gene regions of respiratory pathogens and certain common drug resistance-related sites. The amplified products are purified to construct sequencing libraries. After passing quality control, the libraries undergo paired-end sequencing (2 × 150 bp) using the Illumina MiniSeq platform (MiniSeq High Output Kit, Illumina, United States). Target pathogens included: parainfluenza virus types 1 and 3 (HPIV-1, HPIV-3), coronavirus (229E, HKU1, NL63, and OC43), influenza A (FluA) and B (FluB) viruses, rhinovirus (HRV), adenovirus (HAdV), human metapneumovirus (HMPV), enterovirus (EV), respiratory syncytial virus (RSV), bocavirus (HBoV), herpes simplex virus type 1 (HSV-1), human herpesvirus 6 (HHV-6), human herpesvirus 7 (HHV-7), Epstein–Barr virus (EBV), cytomegalovirus (CMV), *Haemophilus influenzae* (*H. influenzae*), *Streptococcus pneumoniae* (*S. pneumoniae*), *Staphylococcus aureus* (*S. aureus*), *Bordetella pertussis* (*B. pertussis*), *Chlamydophila pneumoniae* (*C. pneumoniae*), *Mycoplasma pneumoniae* (*M. Pneumonia*), *Legionella pneumophila* (*L. pneumophila*), *Acinetobacter baumannii* (*A. baumanni*), *Pseudomonas aeruginosa* (*P. aeruginosa*), *Klebsiella pneumoniae* (*K. peneumoniae*), *Moraxella catarrhalis* (*M. catarrhalis*), *Escherichia coli* (*E. coli*), *group A and B streptococcus* (GAS, GBS), *Candida albicans* (*C. albicans*), and *Aspergillus* (*Aspergillus*). To ensure reliability, all tests are subjected to internal laboratory quality controls and remain within controlled parameters.

### 2.3 Data analysis

Data organization was performed using Excel (2021), and data analysis was conducted with R (v.4.4.1) and GraphPad Prism (v.10.1.2) for statistical analysis. Joinpoint regression analysis, a software developed by the National Cancer Institute (NCI), was utilized for trend analysis of incidence and mortality rates (20), encompassing data processing and visualization operations. Categorical variables were presented as n (%). Intergroup comparisons were performed using the χ^2^ test, with a *p*-value less than 0.05 considered statistically significant.

## 3 Results

### 3.1 Basic characteristics and sample distribution

From 2021 to 2023, a total of 8,533 cases of ARI patients’ screening data were collected, among which 627 cases were excluded due to incomplete data records or insufficient sample size, ultimately incorporating specimens from 7,906 patients for analysis (Table 1). Males accounted for 59.37%, and females for 40.63%; a total of 7,367 cases were actually detected, with males and females accounting for 59.59 and 40.41%, respectively. The results showed that patients under 18 years old accounted for 74.58% of all ARI cases detected, with the highest proportion being those under 5 years old (56.81%). This indicates that the detection rate of respiratory infections gradually decreases with age.

**Table 1:** Summary of demographic characteristics of ARI.

Characteristics	2021	2022	2023	χ^2^	*p*	All cases	Positive	χ^2^	*p*
Total (N)	1,653	2,049	4,204			7,906	7,367		
Sex				0.7662	0.6818			0.07481	0.7845
Male	989	1,228	2,477			4,694	4,390		
Female	664	821	1,727			3,212	2,977		
Age				448.4	<0.0001***			13.39	0.0095**
0 ≤ age < 2	575	407	1,262			2,244	2,109		
2 ≤ age < 5	492	383	1,242			2,117	2,076		
5 ≤ age < 18	314	414	623			1,351	1,309		
18 ≤ age < 65	193	539	537			1,269	1,044		
≥65	79	306	540			925	829		
Monthly				3,816	<0.0001***			412.6	<0.0001***
1	12	306	186			504	473		
2	10	69	263			342	302		
3	72	99	510			681	610		
4	80	67	527			674	650		
5	70	70	621			761	727		
6	99	139	748			986	948		
7	171	194	962			1,327	1,262		
8	118	185	381			684	614		
9	182	161	6			349	304		
10	283	292	0			575	522		
11	271	336	0			67	564		
12	285	131	0			416	391		

The table presents basic information about the study subjects, including age, gender, month, and year of medical visit.

* indicates *p* < 0.05, ** indicates *p* < 0.01, and *** indicates *p* < 0.001, which is considered statistically significant (highlighted in bold).

In terms of seasonal distribution, the largest number of samples was collected from May to August each year, and the detection rate also peaked during this period. The highest number of detections was in summer (2,325 cases, accounting for 31.56%), followed by autumn (2,180 cases, 29.59%), winter (1,477 cases, 20.05%), and spring (1,385 cases, 18.80%). This indicates that the occurrence of ARI has a distinct seasonal pattern, with summer being the season of high detection.

### 3.2 The detection rate and distribution of pathogens in different age groups

**Figure 1:**
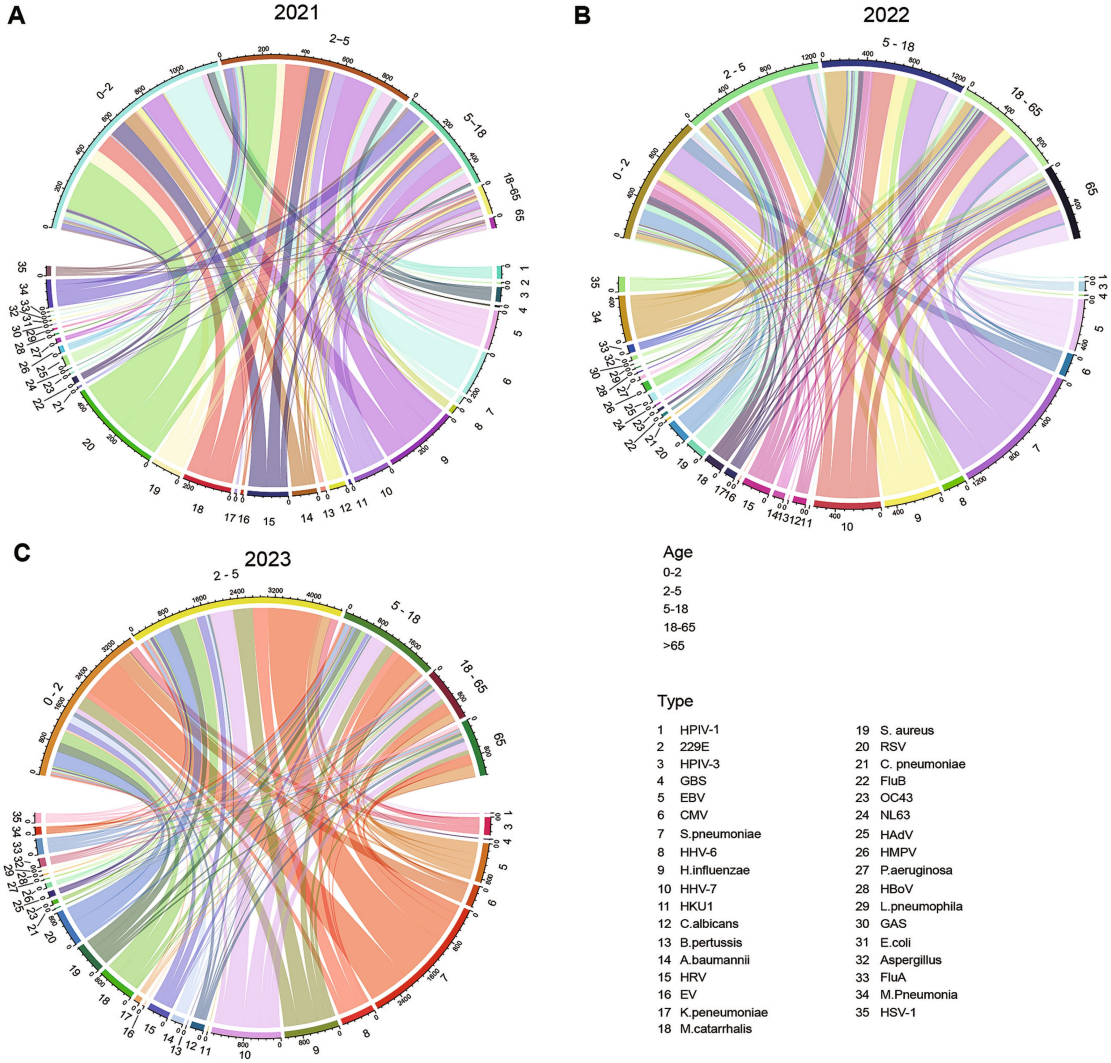
Distribution of detection rates of major ARI pathogens by age group from 2021 to 2023. **(A)** Detection rates of major ARI pathogens by age group in 2021; **(B)** Detection rates of major ARI pathogens by age group in 2022; **(C)** Detection rates of major ARI pathogens by age group in 2023. Three circular chord diagrams labeled **A** (2021), **B** (2022), and **C** (2023) show relationships between age groups and infection types. Each diagram uses colored arcs for age groups and infectious agents, with connecting bands representing case counts across years. A legend identifies age group ranges and infection type labels for reference.

An age stratified analysis of the pathogen detection rate in ARI cases from 2021 to 2023 was conducted (Figure 1). In 2021, the age group with the highest pathogen detection rate was 0–2 years (41.73%), with the main pathogens being RSV (9.25%), CMV (6.90%), and Hib (3.91%). This was followed by the 2–5 years age group (33.00%), primarily characterized by RSV (6.02%), Hib (5.24%), and *M. catarrhalis* (4.01%). The detection rate in the 5–18 age group was 18.23%, primarily consisting of Hib (3.03%), *M. pneumoniae* (2.43%), and HHV-7 (2.36%). The detection rates were lower in the 18–65 age group (5.14%) and the ≥65 age group (1.90%), with the dominant pathogens being EBV (1.06 and 0.74%) respectively.

In 2022, the pathogen detection rate reached its highest in the 5–18 age group (23.73%), with *S. pneumoniae* (5.57%), HHV-7 (3.74%), and *M. pneumonia* (3.72%) being the predominant pathogens. This was followed by the 2–5 age group (23.10%) and the 0–2 age group (21.99%), with *S. pneumoniae* (6.26 and 4.85%) as the main pathogen in each, respectively. The detection rate in the ≥65 age group (12.94%) was higher than in 2021, with EBV (2.83%) and SP (2.37%) as the primary pathogens. *S. pneumoniae* was the dominant pathogen across all age groups, with its positivity rate peaking during the 2–18 age period and subsequently declining gradually.

In 2023, the highest pathogen detection rate was observed in the 2–5 years age group (35.78%), with the main pathogens being *S. pneumoniae* (8.71%), HHV-7 (4.08%), and *H. influenzae* (3.42%). This was followed by the 0–2 years age group (28.15%), where *S. pneumoniae* (7.01%), RSV (2.73%), and CMV (2.48%) were predominant. The pathogen detection rates in the 5–18 years age group (16.99%), ≥65 years age group (9.84%), and 18–65 years age group (9.25%) were relatively lower, with *S. pneumoniae*, HHV-7, and EBV being the main pathogens, respectively. The detection rate of *S. pneumoniae* was highest in the 0–5 years age group and then gradually declined; the detection rate of HHV-7 remained consistently high in those aged 2 years and above.

The comprehensive three-year data show that the pathogen detection rate is highest among preschool children (0–5 years old group), accounting for 60.59% of all detected samples. The main pathogens include *S. pneumoniae* (12.56%), RSV (6.43%), and *H. influenzae* (5.87%).

### 3.3 Monthly pathogen detection rates and trend analysis

**Figure 2:**
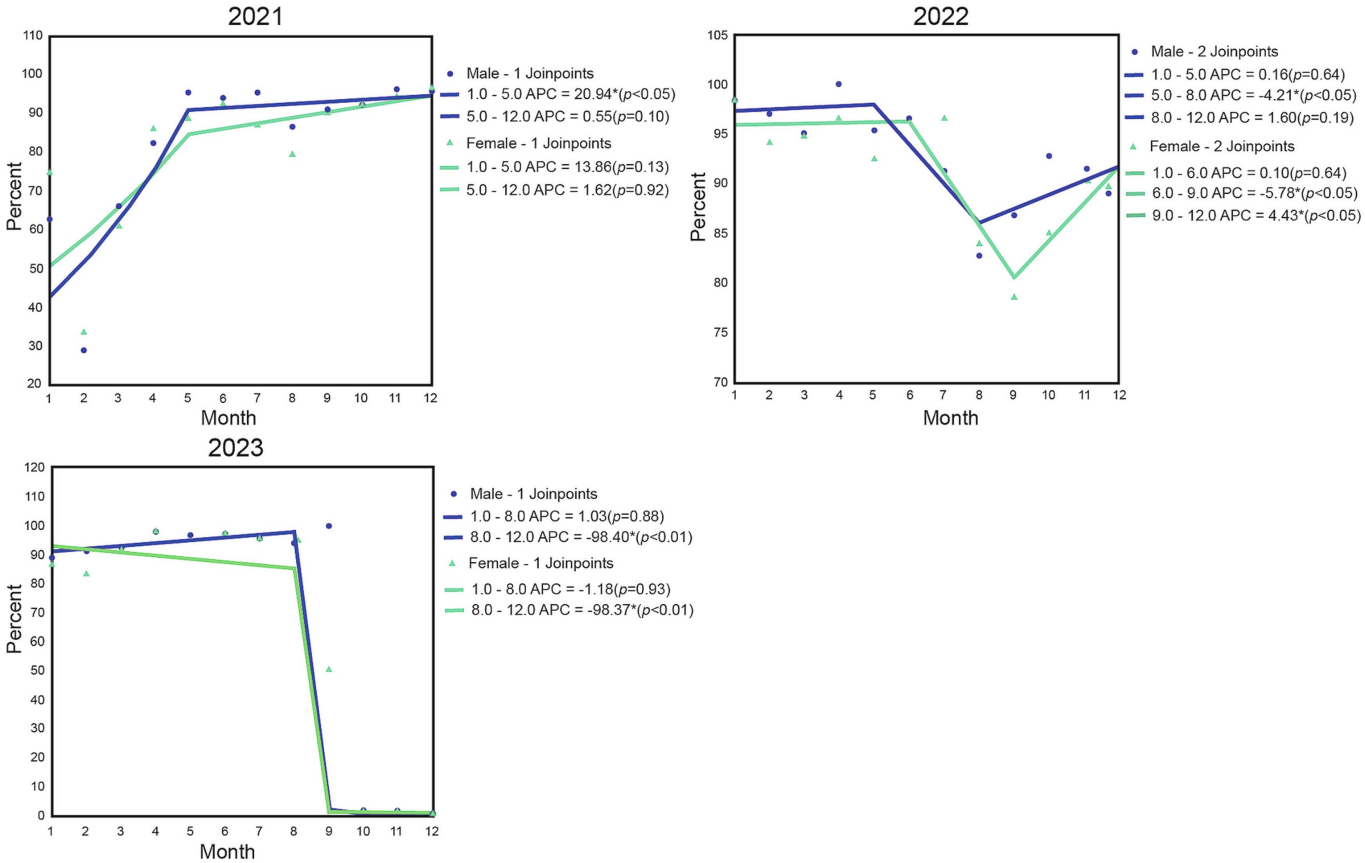
Gender-stratified regression analysis of monthly positive rates of respiratory pathogens from 2021 to 2023. Three line graphs illustrate the monthly percent values for males and females from 2021 to 2023, showing trends, joinpoints, and annual percent change (APC) with statistical values for each segment. Each graph uses blue for males and green for females, with notable increases or decreases at specific months and strong drops in late 2022 and 2023.

Joinpoint regression analysis was conducted to examine the monthly changes in pathogen detection rates among different gender groups from 2021 to 2023 (Figure 2). In 2021, the detection rate remained stable from January to April, with a significant turning point in May, followed by a month-by-month increase, reaching the annual peak by the end of the year. In terms of gender distribution, the detection rate for females was slightly higher than that for males at the beginning of the study. However, starting from May, the detection rate for males surpassed that for females and remained at a higher level thereafter.

In 2022, the detection rate exhibited a “biphasic fluctuation” trend. The positive detection rate for males was higher than that for females throughout the year. From January to April, the detection rate gradually declined, with the first turning point occurring between May and June, followed by a slight rebound from July to August. Unlike in 2021, the detection rate reached its annual low between September and October.

In 2023, the detection rate generally showed an upward trend. From January to June, the detection rate gradually increased, particularly more significantly among the male population.

### 3.4 The detection rates and distribution patterns of pathogens among males and females in different regions

From 2021 to 2023, the detection rates of pathogens in 11 regions of Yunnan Province were predominantly higher in Kunming, Dali, Zhaotong, and other areas (Supplementary Figure S1). While comparing the distribution differences of pathogens across different genders and regions, we further integrated the overall detection frequency of pathogens to comprehensively present the gender and regional disparities.

**Figure 3:**
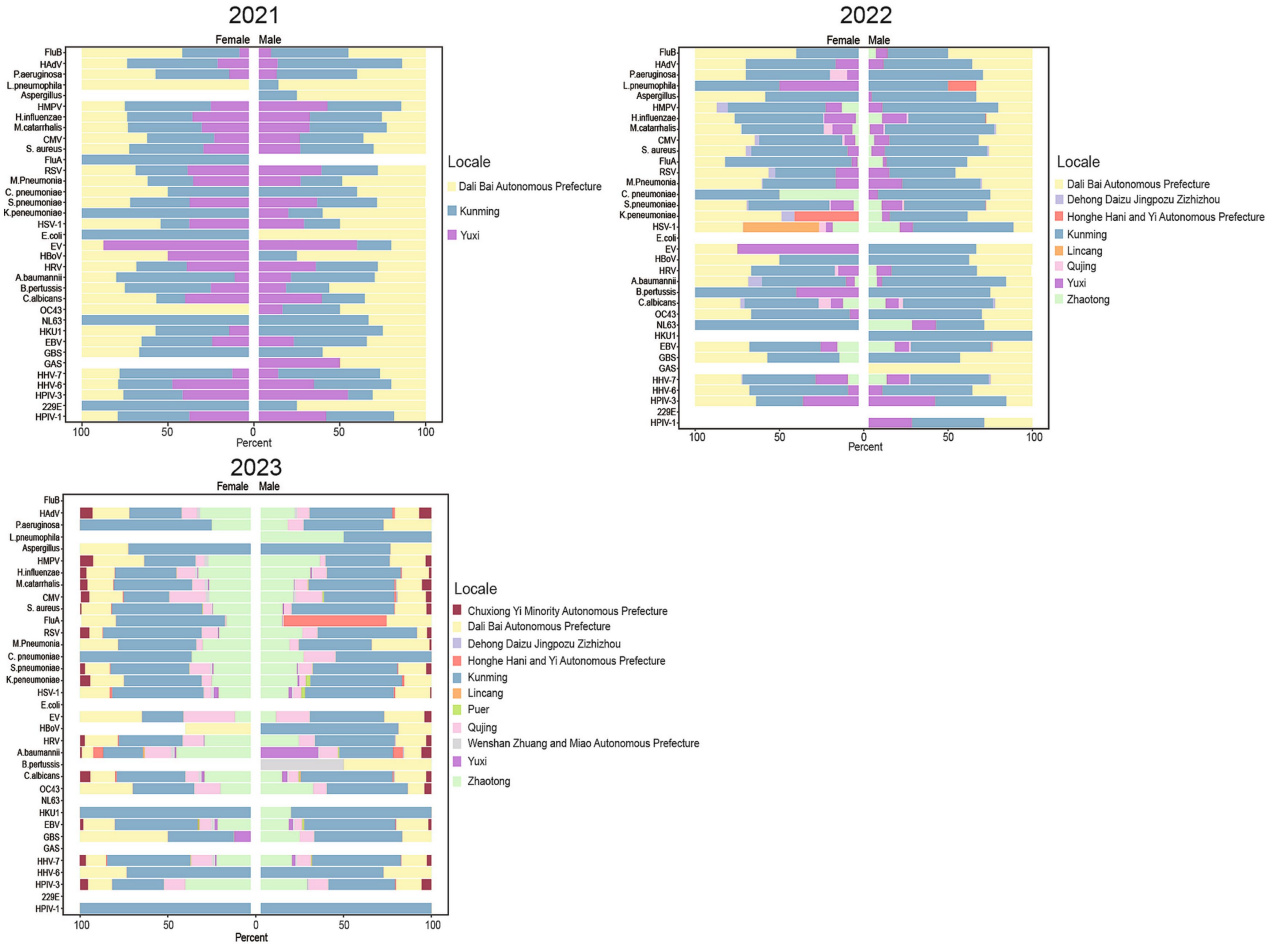
Comparative analysis of ARI pathogen detection rates by region and gender in Yunnan Province from 2021 to 2023. Grouped horizontal bar charts display the gender distribution for multiple pathogens across three years (2021, 2022, 2023) and various locales, with data separated by male and female percentages and color-coded by region for each pathogen.

In 2021, the detection rates of *L. pneumophila*, *E. coli*, *K. pneumoniae*, NL63, and 229E were slightly higher in males than in females, while EBV, CMV, and *S. pneumoniae* showed a more balanced distribution (Figure 3). During this year, the most frequently detected pathogens in both male and female patients were *S. pneumoniae*, *H. influenzae*, and HHV-7, with their detection frequencies significantly higher than other pathogens, forming the primary pathogenic spectrum basis of ARI.

In 2022, the detection rates of HAdV, HBoV, HHV-6, and HPIV-3 were similar between males and females, and their primary distribution areas were also consistent. In the Kunming region, the detection rate of NL63 coronavirus was significantly higher in male patients than in females; in the Yuxi region, the detection rate of EV-G was higher in male patients; in the Lincang region, the detection rate of HSV-1 herpes simplex virus was higher in males; and in the Honghe region, the detection rate of *K. pneumoniae* was markedly higher in males than in females. Additionally, in the Honghe, Kunming, and Dali regions, the detection rates of *L. pneumophila*, HKU1, and GAS showed specific increases in males (Figure 3). Despite the aforementioned gender and regional differences, the most frequently detected pathogens in both male and female patients in 2022 remained primarily *S. pneumoniae*, *H. influenzae*, and HHV-7.

In 2023, the distribution trends of bacterial pathogen detection rates were generally consistent between males and females. However, *L. pneumophila* was predominantly found in female patients, while *B. pertussis* was mainly detected in male patients. The detection rates of other pathogens (such as EB virus and *S. pneumoniae*) were relatively balanced between male and female patients (Figure 3). The most frequently detected pathogens in both male and female patients remained *S. pneumoniae*, *H. influenzae*, and HHV-7. Notably, the aforementioned gender and regional differences were primarily observed among pathogens with high or moderate overall detection frequencies, while no significant gender or regional disparities were evident in low frequency pathogens.

Further analysis of the pathogen detection rates in Kunming, Dali, and Yuxi over 3 years revealed that *K. pneumoniae* and FluB exhibited higher detection rates in these regions, whereas human coronavirus 229E, GAS, and *E. coli* showed lower detection rates. Notably, HHV-6 demonstrated an overall peak in detection rates across all three regions. Additionally, HHV-7 exhibited an increasing trend annually in Kunming, while *A. baumannii* showed a decreasing trend in Dali. Moreover, the detection rate of *B. pertussis* was relatively higher in males than in females in Dali. Overall, *S. pneumoniae, H. influenzae*, and HHV-7 showed higher detection rates in males compared to females over the three-year period.

Regarding specific pathogens, rhinovirus had a relatively higher detection rate in Kunming, EV-G showed a higher detection rate in Yuxi, and RSV displayed a significant upward trend in Kunming over the 3 years. Moreover, the detection rate of *C. pneumoniae* in Kunming is significantly higher than that in other regions (Supplementary Figure S2). Overall, the differences in pathogens across genders and regions primarily manifested as variations in relative detection proportions against the background of common high frequency pathogens, rather than fundamental changes in pathogen composition.

### 3.5 Pathogen co-detection status

**Figure 4:**
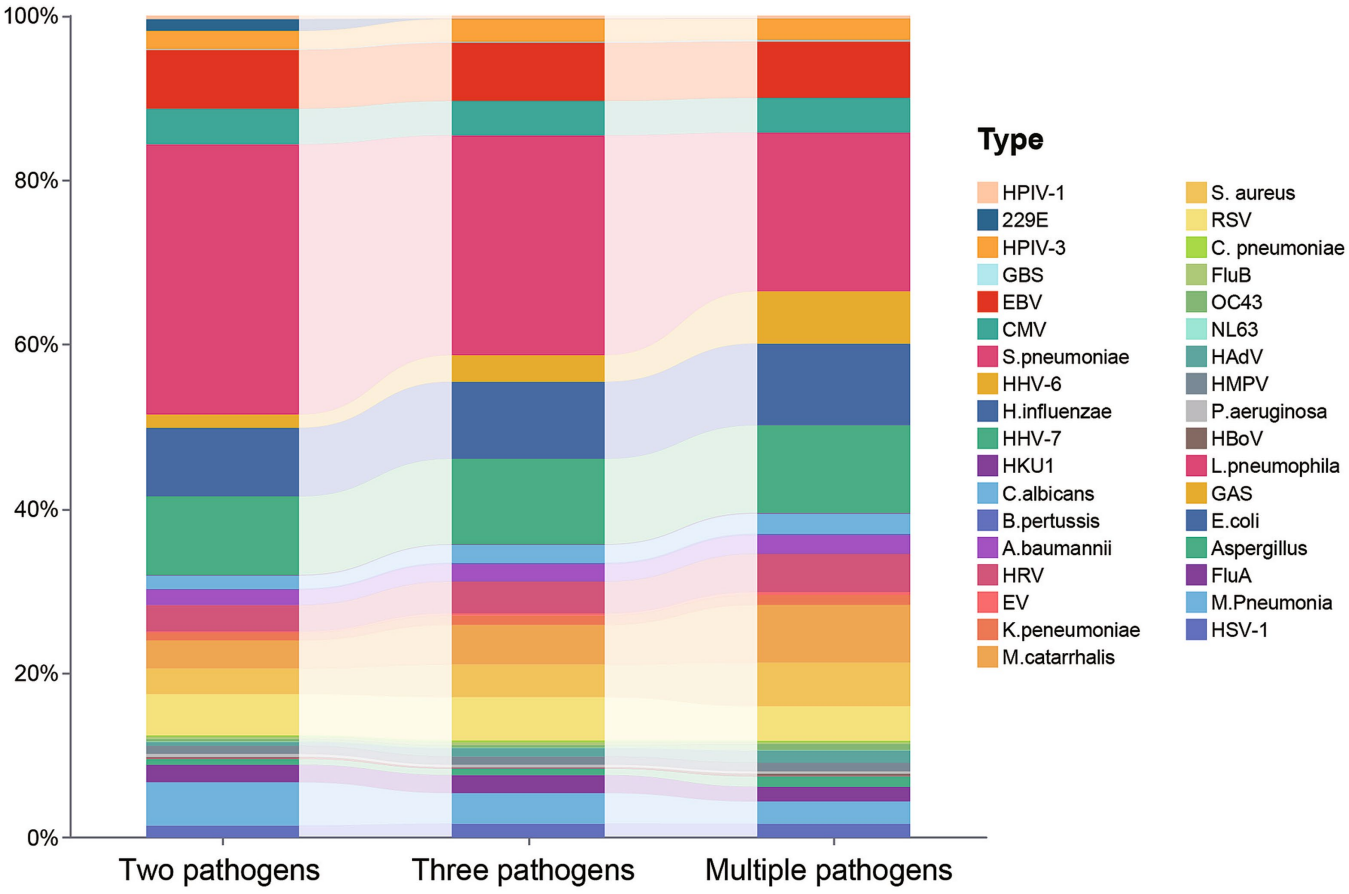
Sankey diagram of ARI pathogen co-detection patterns. Stacked bar chart visualizes the relative frequencies of different pathogen types involved in cases with two, three, or multiple pathogen detections. Each color represents a specific pathogen, as identified in the legend to the right. Proportions shift slightly among groups, indicating changes in pathogen composition with increasing numbers of detected pathogens.

The pathogens with the highest detection rates were *S. pneumoniae*, HHV-7, EBV, *H. influenzae*, and RSV, among others (Table 2). In single detections, *S. pneumoniae* had the highest detection rate (41.89%), followed by HHV-7 (9.35%). In co-detection patterns involving two or more pathogens, the primary pathogens with high detection rates were *S. pneumoniae* (23.57%), HHV-7 (10.41%), *H. influenzae* (9.51%), and EBV (6.97%) (Figure 4). It is noteworthy that the detection rate of *S. pneumoniae* is significantly higher in single detection than in co-detection, whereas HHV-6 exhibits the opposite trend, with its detection rate in co-detection being higher than in single detection.

**Table 2:** Summary of pathogen detection in ARI.

Pathogen	Total infection rate, *N* (%)	Single infection, *N* (%)	Co-infection, *N* (%)
HPIV-1	75 (0.34)	3 (0.26)	72 (0.35)
229E	55 (0.25)	1 (0.09)	54 (0.26)
HPIV-3	557 (2.54)	21 (1.85)	536 (2.58)
GBS	34 (0.16)	0 (0.00)	34 (0.16)
EBV	1,544 (7.05)	96 (8.47)	1,448 (6.97)
CMV	920 (4.20)	43 (3.79)	877 (4.22)
*S. pneumoniae*	5,372 (24.52)	475 (41.89)	4,897 (23.57)
HHV-6	989 (4.51)	4 (0.35)	985 (4.74)
*H. influenzae*	2,041 (9.32)	65 (5.73)	1,976 (9.51)
HHV-7	2,268 (10.35)	106 (9.35)	2,162 (10.41)
HKU1	19 (0.09)	2 (0.18)	17 (0.08)
*C. albicans*	488 (2.23)	23 (2.03)	465 (2.24)
*B. pertussis*	35 (0.16)	4 (0.35)	31 (0.15)
*A. baumannii*	487 (2.22)	35 (3.09)	452 (2.18)
HRV	906 (4.14)	37 (3.26)	869 (4.18)
EV	65 (0.30)	1 (0.09)	64 (0.31)
*K. pneumoniae*	250 (1.14)	13 (1.15)	237 (1.14)
*M. catarrhalis*	1,234 (5.63)	21 (1.85)	1,213 (5.84)
*S. aureus*	981 (4.48)	27 (2.38)	954 (4.59)
RSV	1,003 (4.58)	47 (4.14)	956 (4.60)
*C. pneumoniae*	45 (0.21)	4 (0.35)	41 (0.20)
FluB	56 (0.26)	2 (0.18)	54 (0.26)
OC43	120 (0.55)	4 (0.35)	116 (0.56)
NL63	14 (0.06)	3 (0.26)	11 (0.05)
HAdV	249 (1.14)	2 (0.18)	247 (1.19)
HMPV	222 (1.01)	11 (0.97)	211 (1.02)
*P. aeruginosa*	68 (0.31)	4 (0.35)	64 (0.31)
HBoV	37 (0.17)	0 (0.00)	37 (0.18)
*L. pneumophila*	18 (0.08)	4 (0.35)	14 (0.07)
GAS	3 (0.01)	0 (0.00)	3 (0.01)
*E. coli*	3 (0.01)	0 (0.00)	3 (0.01)
*Aspergillus*	223 (1.02)	3 (0.26)	220 (1.06)
FluA	432 (1.97)	29 (2.56)	403 (1.94)
*M. pneumonia*	735 (3.36)	25 (2.20)	710 (3.42)
HSV-1	359 (1.64)	19 (1.68)	340 (1.64)
*χ*2	/	320.9	
*p*	/	< 0.01***	

The table displays the distribution patterns of single pathogen detection and co-detection of multiple pathogens. The different colors in the figure represent different regions, showing the differences in detection rates of major pathogens among male and female populations across regions.

*p* < 0.05 is marked with *, *p* < 0.01 with **, and *p* < 0.001 with ***, indicating statistical significance (bolded).

## 4 Discussion

ARI is a significant global public health issue, characterized by high incidence rates and potential severe consequences (21, 22). Although there have been relevant epidemiological reports from multiple provinces in China (23–27), due to the unique geography, climate, and population structure of Yunnan Province, regional data remain relatively scarce. By analyzing the pathogen detection data of ARI from 11 regions in Yunnan Province from 2021 to 2023, the composition and distribution characteristics of ARI pathogens in these 11 regions were assessed, along with their associations with age, season, and geographical factors.

Our data showed a higher pathogen detection rate (65.12%) in preschool children aged 0–5 years, consistent with previous studies, further confirming the incomplete development of children’s immune systems and their susceptibility (28–33). The detection rate decreases with age (34), but certain pathogens (such as influenza virus, respiratory syncytial virus, etc.) can still cause severe cases in adult and older adult populations (35, 36), suggesting that stratified prevention and control strategies should be formulated based on age, with a focus on strengthening childhood vaccination, high risk screening, and health education for the older adults.

Research has found that the incidence of ARI in Yunnan Province peaks during the summer months (May to August), differing from the epidemic patterns observed in some regions such as Shaanxi, Macao, and Lanzhou, where the incidence is higher in winter (27, 37, 38). However, it is similar to the patterns seen in Xiamen, other areas of Yunnan, and India, where the incidence peaks during the summer and autumn (39–42). Therefore, seasonal variations may be closely related to Yunnan’s geographical location, climatic characteristics (such as hot and rainy summers), and population behavior patterns (such as children frequently engaging in outdoor activities during summer) (5, 43–45). This suggests that summer should be considered the key season for local prevention and control efforts, requiring advance resource allocation and enhanced surveillance and intervention.

From a geographical distribution perspective, *S. pneumoniae* is the dominant pathogen in all regions, demonstrating its high pathogenicity and widespread prevalence (46, 47). Regional characteristics were also observed. For instance, the detection rate of RSV in Kunming has shown an increasing trend year by year, while in Yuxi, the detection rate of EV is significantly higher, suggesting that local epidemic dynamics are influenced by factors such as population mobility, environmental, and sanitary conditions (48). Gender analysis revealed that the overall detection rate in females is higher than in males, which differs from reports in Shandong and Shaanxi (27, 49). This difference may be related to pregnancy-associated or hormone-related immune suppression in females (50, 51). Meanwhile, the detection rates of certain pathogens (such as *L. pneumophila*, *E. coli*, and *K. pneumoniae*) are higher in males, while an increase in the detection of *L. pneumophila* among females in 2023 reflects the complex role of gender in pathogen transmission. These region-specific characteristics not only contribute to a deeper understanding of the transmission patterns of pathogens but also provide an important basis for formulating localized prevention and control strategies.

Given the significant challenges and lack of uniform standards in defining infection, colonization, and contamination in clinical practice, this study employs the term “co-detection” to objectively reflect the coexistence of multiple pathogens in specimens (52). In the co-detection pattern, *S. pneumoniae* and HHV-7 were found to be the most commonly co-detected pathogens, suggesting possible synergistic infections or shared host susceptibility. The most frequently co-detected pathogens were primarily *S. pneumoniae*, *H. influenzae*, *M. pneumoniae*, and HRV, which may be related to different regions (53). Additionally, the detection rate of HHV-6 in co-detected patients was significantly higher than in single detection cases, indicating that it may be more inclined to function in an environment with multiple pathogen co-detections. It is noteworthy that the detection of HHV-6 was predominantly concentrated in the 0–18 years age group (Figure 1), further highlighting the high susceptibility of children and adolescents to the detection of multiple pathogens (54). This result underscores the need to pay special attention to the co-detection of multiple pathogens in patients of this age group during clinical diagnosis and treatment, and to strengthen the detection and management of rare pathogens such as HHV-6 to avoid missed or misdiagnosis. It should be noted that co-detection of viruses, bacteria, and fungi is more likely to progress to severe disease (52). It is important to note that a positive molecular detection result for viruses, bacteria, or fungi does not necessarily equate to clinical infection, as some results may represent colonization or exogenous contamination. Therefore, clinical diagnosis should be made in conjunction with medical history, imaging, and other laboratory indicators (55, 56).

In summary, this study, based on surveillance data from 11 regions in Yunnan Province from 2021 to 2023, reveals the distribution characteristics and co-detection patterns of ARI pathogens across different age groups, seasons, and geographical areas, providing crucial evidence for understanding regional epidemic trends of respiratory pathogens in the province. The findings reflect both global commonalities and regional specificities. The limitations of this study include reliance solely on laboratory testing without incorporating clinical manifestations, vaccination history, or immune status information, as well as the exclusion of SARS-CoV-2 from pathogen surveillance, which somewhat restricts a comprehensive depiction of the overall respiratory pathogen ecology. During the study, SARS-CoV-2 was managed by an independent COVID-specific surveillance system, and the conventional ARI multi-pathogen detection panel used to generate the retrospective data in this study did not cover this virus, so a unified analysis with other respiratory pathogens could not be achieved methodologically. Nevertheless, this study provides robust data support for understanding the epidemiological characteristics of ARI in Yunnan Province in a non-COVID context, and offers references for improving pathogen surveillance and identifying high risk populations and epidemic seasons. It is recommended to optimize vaccination timing for RSV, influenza, and other diseases based on age and seasonal characteristics, with children as the priority group to complete vaccination before the summer peak. In the future, efforts should be intensified to enhance monitoring and prospective research, promoting the transformation of public health strategies from a “one-size-fits-all” approach to “targeted and forward-looking” measures, so as to reduce the burden of ARI diseases.

## 5 Conclusion

This study, based on the pathogen detection results of 7,906 ARI patients from 11 regions in Yunnan Province from 2021 to 2023, found that ARI exhibits significant distribution differences across various populations and in temporal and spatial dimensions. Preschool children had the highest pathogen detection rate, indicating that they are a key target population for prevention and control. Summer is the main peak season for the epidemic, necessitating enhanced seasonal monitoring and intervention. Regional analysis revealed that *S. pneumoniae* and *H. influenzae* are the dominant pathogens in different areas, highlighting their central role in ARI prevention and control. Additionally, commonly co-detected pathogens such as *S. pneumoniae* and HHV-7 suggest that the coexistence of multiple pathogens is prevalent in ARI. Overall, this study reveals the age, seasonal, and regional specific characteristics of ARI in Yunnan Province, providing important references for the identification of high risk populations, the understanding of epidemic patterns, and the formulation of regionalized prevention and control strategies.

## Data Availability

The original contributions presented in the study are included in the article/Supplementary material, further inquiries can be directed to the corresponding authors.

## Glossary

ARI: Acute respiratory infection
PCR: Polymerase chain reaction
HPIV-1: Parainfluenza virus type 1
HPIV–3: Parainfluenza virus type
229E: Coronavirus 229E
HKU1: Coronavirus HKU1
NL63: Coronavirus NL63
OC43: Coronavirus OC43
FluA: Influenza A virus
FluB: Influenza B virus
HRV: Rhinovirus
HAdV: Adenovirus
HMPV: Human metapneumovirus
EV: Enterovirus
RSV: Respiratory syncytial virus
HBoV: Bocavirus
HSV-1: Herpes simplex virus type 1
HHV-6: Human herpesvirus 6
HHV-7: Human herpesvirus 7
EBV: Epstein–Barr virus
CMV: Cytomegalovirus
*H. influenzae*: *Haemophilus influenzae*
*S. pneumoniae*: *Streptococcus pneumoniae*
*S. aureus*: *Staphylococcus aureus*
*B. pertussis*: *Bordetella pertussis*
*C. pneumoniae*: *Chlamydia pneumoniae*
*M. pneumonia*: *Mycoplasma pneumoniae*
*L. pneumophila*: *Legionella pneumophila*
*A. baumannii*: *Acinetobacter baumannii*
*P. aeruginosa*: *Pseudomonas aeruginosa*
*K. peneumoniae*: *Klebsiella pneumoniae*
*M. catarrhalis*: *Moraxella catarrhalis*
*E. coli*: *Escherichia coli*
GAS: Group A streptococcus
GBS: Group B streptococcus
*C. albicans*: *Candida albicans*
*Aspergillus*: *Aspergillus*
