# Supplementary material for: Epidemiological analysis of respiratory pathogen detection in 11 regions of Yunnan from 2021 to 2023
Source: Front Public Health. 2026 Feb 18;14:1771339. doi: 10.3389/fpubh.2026.1771339 (PMC12957258; doi:10.3389/fpubh.2026.1771339)
Supplement: Supplementary file 1 [file Table_1.DOCX]

Supplementary Material

Epidemiological Analysis of Respiratory Pathogen Detection in 11 Regions of Yunnan from 2021 to 2023.

## Supplementary Figures

**Supplementary Figure 1.** Sampling in various regions of Yunnan Province.

**Supplementary Figure 2.** Association analysis of different genders and the detection rate of each pathogen in three regions from 2021 to 2023.
